# The Impact of Exclusive Enteral Nutrition (EEN) on the Gut Microbiome in Crohn’s Disease: A Review

**DOI:** 10.3390/nu9050447

**Published:** 2017-05-01

**Authors:** Amber MacLellan, Jessica Connors, Shannan Grant, Leah Cahill, Morgan G. I. Langille, Johan Van Limbergen

**Affiliations:** 1 Division of Pediatric Gastroenterology and Nutrition, IWK Health Centre, Halifax, NS B3K 6R8, Canada; Amber.Maclellan@dal.ca (A.M.); Jessica.Connors@iwk.nshealth.ca (J.C.); Leah.Cahill@dal.ca (L.C.); 2 Department of Medicine, Dalhousie University, Halifax, NS B3H 2Y9, Canada; 3 Department of Applied Human Nutrition, Mount Saint Vincent University, Halifax, NS B3M 2J6, Canada; Shannan.Grant2@msvu.ca; 4 Department of Pharmacology, Dalhousie University, Halifax, NS B3H 4R2, Canada; morgan.g.i.langille@dal.ca; 5 Division of Pediatric Gastroenterology and Nutrition, Department of Pediatrics, Dalhousie University, Halifax, NS B3K 6R8, Canada; 6 Department of Immunology & Microbiology, Dalhousie University, Halifax, NS B3K 6R8, Canada

**Keywords:** exclusive enteral nutrition, Crohn’s disease, microbiome

## Abstract

Crohn’s disease (CD), a form of inflammatory bowel disease (IBD), is thought to arise from a complex interaction of genetics, the gut microbiome, and environmental factors, such as diet. There is clear evidence that dietary intervention is successful in the treatment of CD—exclusive enteral nutrition (EEN) is able to induce remission in up to 80% of CD patients. While the mechanism of action of EEN is not clear, EEN is known to cause profound changes in the gut microbiome. Understanding how EEN modifies the gut microbiome to induce remission could provide insight into CD etiopathogenesis and aid the development of microbiome-targeted interventions to guide ongoing dietary therapy to sustain remission. This review includes current literature on changes in composition and function of the gut microbiome associated with EEN treatment in CD patients.

## 1. Introduction

Crohn’s disease (CD) is a chronic relapsing and remitting inflammatory bowel disease (IBD) that causes tissue-damaging inflammation within the gastrointestinal (GI) tract. The exact cause of CD is unclear but has been attributed to complex genetic and environmental factors that interact to trigger abnormal immune responses to commensal gut microbes [1]. Because commensal microbes are thought to play a pivotal role in the development of CD, there is considerable interest in targeting the commensal microbiome for therapeutic purposes [2,3]. Diet, in particular, has emerged as an important lifestyle factor that greatly influences microbiome composition and regular dietary patterns are associated with IBD risk [4,5,6,7,8]. Strict regulation of dietary intake has been shown to induce and, to some degree, maintain remission (i.e., symptom-free disease) in both children and adults with CD [9,10,11].

Perhaps the strongest evidence for the role of diet in CD is the therapeutic effect of exclusive enteral nutrition (EEN) in treating active CD. EEN involves administering a complete liquid diet of formula for 4 to 12 weeks, either orally or via nasogastric tube [12,13]. Formulas can be classified as elemental or polymeric depending on whether the protein source is delivered as individual amino acids or intact protein, respectively. EEN has been shown to be as effective as traditional corticosteroid therapy in pediatric studies, with approximately 80% of CD patients achieving remission [11,12,14,15]. Because adults struggle more with compliance to EEN, studies demonstrating the efficacy of EEN in adult CD patients have been less conclusive [12,16]. Notably, in contrast to steroid use, EEN addresses nutritional deficits associated with CD and is associated with few to no adverse side effects, better growth outcomes in pediatric CD patients, and mucosal healing in the intestine [17,18,19]. Despite the recommendation of EEN as first-line therapy in pediatric CD, it is still not commonly practiced in North America to treat pediatric or adult populations [20,21]. Factors that have been identified as inhibitors of the widespread use of EEN include patient non-adherence, low familiarity among clinicians, and poor understanding of its mode of action in treating CD-associated inflammation [20].

The mechanisms by which EEN reduces both physiological and metabolic markers of inflammation in CD are largely unknown [22]. However, mounting evidence supports that EEN potently modifies microbial communities residing within the gut [23,24]. These microbial communities (collectively referred to as the microbiome) support intestinal homeostasis and the immune system function [25] and it is widely accepted that disruptions in host–microbe interactions are the driving force behind tissue-damaging inflammation in CD [25,26,27]. Thus, if EEN works through a favorable modification of microbial-based gut inflammation, assessing changes in the microbiome during EEN could offer clues to which microbes and/or microbial metabolites play a role in the etiology of CD.

In this review, we have summarized the current literature regarding the impact of EEN on the gut microbiome in CD. It is apparent that EEN is effective and currently the only dietary intervention routinely used to treat CD, although still underutilized and understudied in clinical practice settings [9]. Ultimately, a better understanding of how EEN works will help promote its use in CD treatment and potentially aid the development of new diet-based therapies targeting the microbiome.

## 2. Dysbiosis in CD

Characterizing the CD-associated microbiome offers important insight into disease etiology, and also provides an opportunity for evaluating the impact of microbiome-targeted therapies [28]. While several putative bacterial pathogens have been linked to CD, including species from *Mycobacterium*, *Campylobacter*, *Escherichia* and *Helicobacter*, current evidence does not support that CD is caused by a single bacterium [27]. Rather, CD is associated with significant community-level imbalances in the gut microbiome (i.e., dysbiosis), as inferred from stool and/or mucosal biopsy samples. The human gut microbiome is dominated by four major phyla: Firmicutes, Bacteroidetes, Actinobacteria, and Proteobacteria. In healthy individuals, over 90% of species belong to Firmicutes and Bacteroidetes [27]. General features of CD-associated dysbiosis include a reduction in the phyla Firmicutes and an expansion of the phyla Proteobacteria, as well as an overall decrease in bacterial diversity [29]. An important aspect of CD-associated dysbiosis appears to be the loss of symbionts with anti-inflammatory properties [30]. Depletion of Firmicutes, including *Clostridia,* and *Bifidobacterium*, has been documented repeatedly in CD patients [29,31,32]. At the species level, reduced abundance of *Faecalibacterium prausnitzii* is a well-characterized feature of CD. *F. praustnitzii* is a major representative of the *Clostridium leptum* group that produces anti-inflammatory metabolites including short-chain fatty acids (SCFAs) [33,34,35]. Conversely, CD-associated dysbiosis has also been linked to the expansion of potentially pathogenic symbionts or ‘pathobionts’ [36]. These include commensals that have immune-stimulating properties and can overactivate immune responses to cause destructive inflammation. For instance, a *Clostridia*-related group of segmented filamentous bacteria (SFB) has been shown to modulate intestinal inflammation in animal models through a unique capacity to induce Th17 responses [37,38]. While the specific role of SFB in CD is unresolved, overrepresentation of pathobionts including Proteobacteria (*Escherichia coli* and *Enterobacteriaceae*) has been documented in CD patients and may be an important factor compounding the loss of protective symbionts in dysbiotic communities [31,32].

While the existence of CD-associated dysbiosis is well-established, the precise nature of dysbiosis may vary between individuals and study cohorts, and can be influenced by study design and sampling protocols. Many reports to date have relied on opportunistic sampling of patients with established disease, but this approach is limited significantly by confounders such as active treatment and chronic inflammation. Instead, microbiome features with a causal role in disease may be more identifiable in newly-diagnosed treatment-naïve patients. In the largest treatment-naïve CD cohort study to date (447 pediatric CD patients, 221 controls), Gevers et al. identified several taxa differentially associated with disease using mucosal biopsy samples. Specifically, CD was associated with increased abundance of *Enterobacteriaceae*, *Pasteurellaceae*, *Veillonellaceae* and *Fusobacteriaceae*, and decreased abundances of *Bifidobacteriaceae*, *Erysipelotrichales*, *Bacteroidales* and *Clostridiales* [32]. However, this profile was only weakly reflected in stool samples provided by a subset of patients [32]. While CD-associated dysbiosis has been observed using both stool and mucosal biopsy samples throughout the available literature, it is important to note that the fecal microbiome profile is not necessarily reflective of the mucosa-associated microbiome [39,40].

## 3. Molecular Approaches to Microbiome Investigation

Culture-independent DNA-based methods have been widely used to describe and/or phylogenetically identify components of the gut microbiome, since the majority of total gut-resident bacteria are not readily culturable [41]. Most studies, including many reviewed herein, rely on the amplification of the 16S ribosomal RNA (rRNA) gene, a marker gene that is highly conserved among bacteria but variable enough to identify species. The nine variable regions (V1–V9) of the 16S gene are flanked by conserved sequences, enabling polymerase chain reaction (PCR) amplification of variable sequences by ‘universal’ primers and downstream analysis of 16S rRNA amplicons [42].

DNA fingerprinting techniques such as denaturing gradient gel electrophoresis (DGGE) or temperature gradient gel electrophoresis (TGGE) can be used for a general screening and/or comparative analyses of diversity [43]. Briefly, PCR-amplified 16S rRNA genes from the community sample are submitted to electrophoresis in a polyacrylamide gel with an increasing gradient of denaturant or temperature. Distinct banding patterns form based on the characteristics of the 16S rRNA gene sequences in the sample [43]. Terminal restriction fragment length polymorphism (T-RFLP) analysis is a similar approach to microbiome fingerprinting wherein 16S rRNA amplicons are fragmented by restriction enzymes, and then separated by electrophoresis with band visualization [43].

Such fingerprinting tools are surpassed by the depth of analysis available with next-generation sequencing (NGS) technologies. NGS allows high-throughput profiling of the microbiome via targeted 16S rRNA gene sequencing or shotgun metagenomic sequencing followed by comparison to known bacterial sequence databases [44]. In addition, 16S rRNA amplicon sequences are clustered into operational taxonomic units (OTUs) based on shared sequence identity. In contrast to targeted sequencing, shotgun approaches non-selectively sequence the total genomic content (i.e., metagenome) of the sample, which provides valuable information about both the composition and functional capacity of the community [44]. Community-level analysis can be more powerful in detecting overall community shifts than examining individual taxa when there are many small changes in taxa throughout a community.

## 4. Microbiome Changes Associated with EEN

Studies investigating the impact of EEN therapy on the microbiome are limited. Since EEN has been used most consistently in the treatment of pediatric CD, the majority of existing microbiome studies have been conducted in children, and have generally had small sample sizes (see Table 1). Interpreting detailed microbiome changes across different studies is also complicated by the heterogeneity of study cohorts, high inter-individual variation, differences in sample site (i.e., stool versus mucosal biopsy), and methodological differences such as the type of formula and which oral foods, if any, were allowed. Nonetheless, some general patterns have emerged.

### 4.1. Composition and Diversity

DNA fingerprinting studies were the first to report the profound microbiome-modulating effects of EEN therapy in CD. Analyzing banding patterns of 16S rRNA gene amplicons by DGGE, Lionetti et al. observed marked compositional changes over an eight-week period of EEN treatment in nine patients, while samples collected from healthy children over the same period appeared relatively stable [45]. In a similar study, Leach et al. compared the percent similarity of DGGE banding patterns in six children before and after EEN and found significantly greater change in microbiome composition with EEN compared to control samples [46]. Changes were assessed across all bacteria and within key intestinal bacterial groups including *Bifidobacteria*, *Bacteroides-Prevotella*, *Clostridium coccoides* and *C. leptum.* Bacterial diversity (i.e., band count) was not significantly different between controls and CD patients prior to treatment, but CD patients were found to have lower diversity at the end of treatment relative to controls. In particular, the abundance of species belonging to the *Bacteroides-Prevotella* and *C. coccoides* groups were significantly lower in CD patients following EEN. A significant correlation was found between the degree of change in *Bacteroides*/*Prevotella* group bacteria and clinical improvement over the period of EEN treatment [46]. Shiga et al. also observed a significant decline in *Bacteroides fragilis* via T-RFLP analysis in a study of eight CD patients undergoing EEN with elemental formula, although no change in overall diversity (i.e., total number of T-RFs) was detected [47].

In a larger and more recent study involving 15 pediatric CD patients and 21 health controls, Gerasimidis et al. observed a marked decline in diversity during EEN treatment through TGGE banding patterns [48]. The authors also used real-time quantitative PCR (qPCR) to quantify bacterial groups of interest and, similar to Leach et al., found a decrease *Bacteroides/Prevotella* group species in children who showed clinical improvement [46,48]. Notably, Gerasimidis et al. reported a significant decrease in *F. prausnitzii* with EEN treatment, which appears to contradict previous associations of this bacterium with positive clinical outcomes in adult CD [33,48,49]. Reduced abundance of *F. prausnitzii* (specifically, two subgroups of *F. prausnitzii*) was also reported to correlate with clinical improvement with EEN treatment in an adult CD study by Jia et al. [50]. The paradoxical decrease of *F. prausnitzii* in correlation with improvement in clinical symptoms indicates that the therapeutic effect of EEN is not mediated by *F. prausnitzii*. However, the physiological significance of *F. prausnitzii* levels in CD has not been fully established. For instance, a study of biopsy samples from CD patients reported that *F. prausnitzii* was significantly more abundant in patients than controls, suggesting a more complex role for this bacterium [51].

With the advent of modern NGS technologies, the entire microbiome community can be profiled as opposed to targeting specific taxa for analysis. Using 16S rRNA gene NGS, D’ Argenio et al. characterized microbiome changes in the ileal mucosal microbiome in response to EEN in a single CD patient and matched control [52]. Patient dysbiosis prior to EEN therapy was characterized by reduced diversity, and an imbalance of less Bacteroidetes and more Proteobacteria compared to the control, which is consistent with previously reported features of CD-associated dysbiosis. After EEN, diversity and the relative abundances of Bacteroidetes increased and Proteobacteria decreased to more closely resemble the control sample [52]. While these preliminary results suggest that EEN may ‘normalize’ disease-associated dysbiosis to cause remission, it should be noted that this study examined the mucosal microbiome, whereas all other studies on the microbiome in EEN to date have used stool samples.

In a study of five newly-diagnosed children with CD undergoing EEN and five controls, Kaakoush et al. observed decreased microbial diversity in CD patients and a dysbiosis that was highly variable among individuals [53]. EEN treatment resulted in a further decrease in the number of OTUs, which is consistent with previous observations that EEN reduces diversity [46,48]. The study primarily examined EEN-induced changes in six families within the Firmicutes phylum (*Erysipelotrichaceae*, *Ruminococcaceae*, *Lachnospiraceae*, *Streptococcaceae*, *Veillonellaceae* and *Peptostreptococcaceae*) and found, in general, that reduction in their relative abundance correlated with clinical improvement, though changes in specific families varied among study subjects [53]. In contrast, Schwerd et al. reported that EEN treatment increased relative abundance of bacteria within the *Firmicutes* phylum, especially members of the *Christensenellaceae* family, and decreased relative abundance in members of the *Bacteroidetes* phylum (including *Bacteroidaceae*, *Porphyromonadaceae* and *Rikenellaceae*) in eight patients undergoing EEN [54].

In a larger study of 23 pediatric CD patients undergoing EEN, Quince et al. observed that microbiomes of CD patients had lower diversity compared with healthy controls, and showed reduced abundance of classical commensals such as *Faecalibacterium* and *Bifidobacterium*, and less-studied taxa including *Eubacterium rectale* and *Ruminococcus obeum* [55]. EEN further decreased microbiome diversity and caused significant changes in microbiome structure, particularly in those genera that were already reduced in CD patients, such as *Faecalibacterium, Bifidobacterium* and *Ruminococcus;* only *Lactococcus* increased during EEN. Altogether, Quince et al. observed that EEN shifted the microbiome to a more dysbiotic state, as opposed to normalizing it to more closely resemble controls [55]. Notably, this study demonstrated that different species within the same genus can be over- or under-represented in CD patients, highlighting the need for in-depth microbiome analysis.

Lewis et al. recently performed a metagenomic analysis of 26 healthy controls and 86 pediatric CD patients, including 22 patients undergoing EEN. EEN treatment was associated with an initial decrease in *Dialister*, *Dorea*, *Streptococcus*, *Gordinibacter* and *Haemophilius*, and increase in *Alistipes* [56], which are bile-tolerant, amino-acid metabolizing microbes associated with animal-based diets [57]. Increased abundance of *Alistipes* was also observed in a small study of four patients with active CD treated with EEN, accompanied by a decrease in taxa from the *Proteobacteria* phylum [58]. After just one week of treatment, Lewis et al. observed marked differences in microbiome composition between patients who ultimately responded to EEN and those who did not. By eight weeks of EEN, the patients who responded to treatment exhibited profiles closer to healthy controls whereas non-responders were more dissimilar. This observation is consistent with previous reports, including those by Gerasimidis et al. and Leach et al., that microbiome features of patients who respond to EEN are different from those who do not respond [46,48].

Interestingly, Lewis et al. found that their larger cohort of 86 CD patients clustered into two distinct groups before treatment: one closer to healthy controls and one farther or more ‘dysbiotic’ relative to controls. Overall, CD-associated dysbiosis was characterized by reduced diversity and reduced relative abundance of *Prevotella*, *Eubacterium*, *Odoribacter*, *Akkermansia*, *Roseburia*, *Parabacteroides*, *Alistipes*, *Coprococcus*, *Dorea*, *Ruminococcus* and an increased abundance of *Escherichia*, *Klebsiella*, *Enterococcus* and *Veillonella*, with the more dysbiotic cluster having even lower diversity and greater alterations in composition, particularly increased proportions of *Lactobacillus* and reduced proportions of *Blautia*, *Faecalibacterium*, *Dialister* and *Bacteroides* [56]. It is not clear from this study whether patients belonging to the near or far dysbiotic cluster before treatment responded differently to EEN. However, given that responders and non-responders exhibited divergent microbiome changes in response to EEN, the role of pre-treatment dysbiosis in EEN outcomes warrants further investigation.

Evidence from previous studies suggests that microbiome profiling before or during EEN could identify patient subgroups that are most likely to benefit from EEN therapy. In a recent study of ten pediatric CD patients undergoing EEN and five healthy controls, Dunn et al. further investigated this notion by comparing microbiome community structures between patients that maintained remission for at least six months following EEN (sustained remission; SR), and patients who did not achieve or remain in remission (non-sustained remission; non-SR) [59]. Prior to EEN, SR patients had similar relative abundances of taxa within *Verrucomicrobia*, *Firmicutes* and *Bacteroidetes* as healthy controls, whereas non-SR had a strikingly higher abundance of *Proteobacteria*, a commonly-reported feature of CD-associated dysbiosis [29,52,60]. Dunn et al. observed that *Proteobacteria* increased further in non-SR patients over the course of EEN treatment [59]. Furthermore, bacterial diversity decreased during EEN in SR patients, but increased in non-SR patients. Using a novel Bayesian analytical framework to overcome inter-individual variation and identify the microbial-level associations with SR or non-SR status, Dunn et al. found the most predominant OTUs associated with sustained remission to be from *Akkermansia muciniphila*, *Bacteroides* (including *B. fragilis* and *B. ovatus*), *Lachnospiraceae* and *Ruminococceae*. The predominant OTUs associated with non-sustained remission were from *Bacteroides* as well (including *B. plebeius*), *Enterobacteriaceae* (including *Klebsiella*), and *Prevotella* [59].

### 4.2. Metabolic Functions

Microbial metabolic functions are consistently perturbed in CD patients, perhaps even more so than microbiome composition [61,62]. The gut microbiome performs vital metabolic functions that result in the production of bioactive metabolites capable of regulating intestinal physiology and immune function [63]. That being said, examination of functional interactions among microbes within the gut microbiome presents significant analytical challenges due to the large number of species, high inter-individual variation in species composition, and the wide array of metabolic interactions occurring among bacteria and with the host [44,64,65,66,67]. Moreover, functional redundancy in the microbiome—defined as multiple bacterial species performing the same biochemical processes, or generating the same metabolic products—can further complicate analysis [64].

Studies on the impact of EEN on microbiome metabolic functions are limited, but existing data supports that EEN potently modifies the production of microbial metabolites. Walton et al. observed that fecal concentrations of microbial products including SCFAs, as well as potentially toxic chemicals including 1-propanol, the methyl and ethyl esters of SCFAs, decreased significantly in CD patients after a two week course of EEN [68]. EEN was also associated with increased breath concentrations of phenol and indole, which is consistent with a switch from fermentation of complex carbohydrates to protein-based metabolism [68]. Similarly, Gerasimidis et al. observed significantly decreased concentrations of fecal butyrate over the course of EEN (likely reflecting the low fibre content of EEN), and an increase in fecal sulfide and pH levels [48], which may also reflect protein catabolism [69].

While these studies indicate that EEN impacts microbial metabolism within the gut, they are limited by specific targeting of known bacterial metabolites. In contrast, NGS technologies allow a hypothesis-free assessment of community-level functions. Shotgun metagenomic sequencing reads can be assigned to functional modules through alignment to Kyoto Encyclopedia of Genes and Genomes (KEGG) to identify and reconstruct genes into broader biological pathways [44]. Using this approach, Quince et al. found significant differences in the metabolic potential between CD patients and controls. Despite having low taxonomic diversity, CD patients had a higher level of functional diversity than controls, which may reflect the range of metabolic niches that can be exploited in the inflamed gut microenvironment [55,61]. Modules for ubiquinone and lipopolysaccharide biosynthesis and the twin-arginine translocation system were more abundant in CD patients and may be due to the over-representation of *Enterobacteriaceae*, which includes pathobionts such as *E. coli*. Conversely, healthy controls had a greater capacity for key processes such as fatty acid biosynthesis (initiation) and sulfur reduction. Over the course of EEN, functional diversity tended to decrease to levels comparable to healthy controls, suggesting functional redundancy across multiple species. EEN specifically increased genes involved in the transport of spermidine/putrescine, which have an important role in cell growth and could reflect healing of mucosal epithelial cells. Interestingly, Quince et al. reported that EEN treatment reduced the abundance of *Atopobium parvulum*, which is known to produce hydrogen sulfide (H_2_S) through the fermentation of sulfur-containing amino-acids [55,70]. A recent study by Mottawea et al. identified *A. parvulum* as a prominent microbe controlling a central hub of H_2_S-producing bacterial genera in CD patients [70]. An increased abundance of H_2_S producers in CD patients was associated with defects in H_2_S detoxification, suggesting an important mechanistic role of host-microbe interactions in CD inflammation [70].

In a recent metagenomic analysis of ten pediatric CD patients and five controls, Dunn et al. observed that CD patients tended to have an excess of low abundance modules relative to controls, which could be a reflection of divergent functional capacities. In total, the authors identified eight pathways with significantly different abundances in CD patients. These pathways suggest that CD patients have an increased community-level capacity for degrading environmental pollutants and xenobiotics, and increased metabolic formation of succinate, a metabolite shown to increase intestinal inflammation in rodent models of colitis. Conversely, CD patients had decreased abundance in genes encoding bacterial heat shock proteins, which may influence immune regulation within the intestine [71]. Interestingly, pathway abundance was similar between controls and patients who sustained remission following EEN, whereas patients who did not sustain remission exhibited the most pronounced difference relative to controls. Further investigation incorporating metabolomic data and bile acid profiling will be required to establish the mechanistic connection between altered microbiome metabolic functions and EEN.

## 5. Microbiome Changes Associated with Return to Free Diet

EEN-induced remission in CD is often transient in nature; approximately 60–70% of patients relapse within 12 months of EEN cessation [72,73]. There is limited information on microbiome changes that accompany the return to regular diet following EEN in CD patients. Studies in healthy subjects have shown that while microbiome community structure can change rapidly in response to short-term dietary interventions, the microbiome typically reverts to its prior composition once the intervention ceases [57,74]. Consistent with this notion, Leach et al. found that while microbiome profiles were only 15–38% similar to pre-treatment profiles after eight weeks of EEN, profiles four months after EEN showed 31–41% similarity to pre-treatment profiles, indicating a partial reversion [46]. Likewise, Gerasimidis et al. observed a regression of major EEN-induced microbiome changes upon return to habitual free diet. Specifically, microbiome diversity and *F. prausnitzii* levels, which had been depleted during EEN, increased significantly along with concentrations of fecal SCFAs and sulfide [48].

It is postulated that abstention from regular food, rather than administration of EEN formula itself, is critical to the success of EEN therapy in treating CD [23]. Certain food components common in westernized diets, such as emulsifying agents and certain complex carbohydrates, have been shown to have detrimental effects on intestinal homeostasis [4]. Indeed, commonly-used food additives carrageenan and carboxymethylcellulose (CMC) induce intestinal lesions and inflammation in animal models [75]. Studies comparing EEN to partial EN (i.e., formula plus regular diet) show a clear superiority for EEN to induce remission [76], which supports to some degree that EEN may also work by exclusion. Further investigation is required to examine microbiome changes upon reintroduction of regular diet and to develop evidence-based dietary advice to help sustain remission (e.g., regarding fibre consumption). Ongoing dietary intervention studies will hopefully help to identify food components that may have contributed to the CD-associated dysbiosis and avoidance of these food components during remission may become an adjunct maintenance treatment strategy to avoid triggering microbiome reversion and/or disease flares following cessation of EEN [10].

## 6. Conclusions

The mode of action of EEN to treat CD remains unclear. EEN is a highly efficacious treatment to induce remission in CD patients and has a profound impact on the microbiome, suggesting that EEN positively interferes with dysbiosis in CD patients. The emerging theme from studies published to date is that EEN causes a broad reduction in bacterial diversity, changes community-level metabolic functions, and, at least initially, may increase microbial dysbiosis. The lack of standardized methodology and the high inter-individual variability of microbiome structure across studies have generated some conflicting results concerning specific taxonomic shifts, especially at classifications lower than phylum-level. General taxonomic shifts associated with EEN included: reduced abundance of *Firmicutes* (including *Faecalibacterium*), *Bacteroides/Prevotella*, and *Proteobacteriaceae* and an increase in taxa belonging to *Bacteroidetes* (e.g., *Alistipes*) [46,48,52,56,58,59]. Whereas early studies predicted that EEN treatment might shift dysbiosis toward a healthier state, more recent data indicate that EEN initially induces a microbiome state that is even more dissimilar to healthy controls. This striking observation suggests that EEN perhaps mediates its effect by disrupting established CD-associated dysbiotic microbial communities to allow for re-colonization and establishment of communities that form a more balanced interaction with the host. Recent work has shown that CD patients can have distinct forms or ‘degrees’ of dysbiosis, which could impact the likelihood of achieving remission with EEN alone [56,59,77]. For patients with more severe dysbiosis, the implications could include an early requirement for additional (microbiome modulating) therapy and/or increased duration of EEN treatment to alter the microbiome toward a more beneficial configuration. Importantly, the vast majority of information on microbiome changes associated with EEN has been obtained from stool samples. This is likely a reflection of the practical and ethical challenges in obtaining serial biopsy samples relative to non-invasive stool sample collection. Further investigation is required to improve our understanding of how EEN modulates the mucosal microbiome.

There is an urgent need to understand the connection between diet, the microbiome and disease activity in CD. Patients with CD express strong interest in controlling their disease with diet, but medical nutrition therapy (MNT) is currently not included in standard care for CD due to a lack of research in this area [4,78]. Given that EEN is highly restrictive and not feasible for long-term maintenance [20], targeted elimination diets present a more sustainable and preferable intervention [79]. Defined solid food-based diets based on the exclusion of certain foods have shown potential clinical efficacy in treating CD, but the mechanistic connection between dietary intake and inflammatory disease activity remains poorly defined [10,80,81,82,83,84,85].

A better understanding of these processes could have clinical applications outside of CD treatment. For instance, recent studies show that EEN also has anti-inflammatory and microbiome-modulating effects in the treatment of juvenile idiopathic arthritis [86,87]. There is increasing evidence that diet modulates the microbiome and disease activity in ulcerative colitis (UC), a form of IBD localized to the colon [88,89]. However, robust studies to evaluate the efficacy of EEN have not been feasible due to side effects of liquid diet (i.e., increased stooling frequency). Insight into the mechanisms of EEN in CD could help to inform the development of solid food-based MNT to treat UC. Future studies are required to examine the role of EEN- and diet-induced microbiome changes in modulating intestinal inflammation. Moreover, in the absence of standard medical nutrition therapy, efforts to develop and evaluate patient-focused dietary interventions are warranted.

## Figures and Tables

**Table 1:** Studies of microbiome changes associated with EEN in Crohn’s disease.

Reference	Subjects	Sample Type	Methods	Major Findings Associated with EEN
Lionetti et al. [45] (2005)	9 CD	Stool	PCR-TGGE 16S rRNA gene, V6–V8 region	Decreased diversity
	5 HC			
Leach et al. [46] (2008)	6 CD	Stool	PCR-DGGE 16S rRNA gene using primers for total bacteria (Eubacteria), *Bacteroides-Prevotella*, *C. coccoides*, *C. leptum* and *Bifidobacteria*	Decreased diversity, particularly in *Bacteroides-Prevotella* and *C. coccoides*
	7 HC			
Jia et al. [50] (2010)	20 CD	Stool	PCR using primers against nucleotidyl transferase gene and butyryl-CoA transferase gene in *Faecalibacterium prausnitzii* subgroups A2-165 and M21/2	Decreased/sustained low levels of both *F. prausnitzii* subgroups
Shiga et al. [47] (2012)	8 CD	Stool	PCR-T-RFLP 16S rRNA gene, full length; qPCR 16S rRNA gene for total bacteria, *Bifidobacterium*, *Bacteroides fragilis* group, *Clostridium coccoides* group, *C. leptum* group, *Enterococcus*, *Escherichia coli* and *Lactobacillus*	No change in overall diversity; significant decline in *Bacteroides fragilis*
	17 HC			
D’Argenio et al. [52] (2013)	1 CD	Ileal biopsy	16S rRNA gene, V4–V6 region NGS	Increased diversity; increased Bacteroidetes and decreased Proteobacteria
	1 HC			
Gerasimidis et al. [48] (2014)	15 CD	Stool	qPCR 16S rRNA gene for total bacteria, *Bacteroides-Prevotella*, *Bifidobacterium*, *C. coccoides*, *C. leptum*, *Lactobacillus*, *E. coli*, *F. prausnitzii*	Decreased diversity and decreased *Faecalibacterium prausnitzii*
	21 HC			
Kaakoush et al. [53] (2015)	5 CD	Stool	16S rRNA gene V1–V3 region and shotgun metagenome NGS	Decreased diversity; six families of Firmicutes (*Erysipelotrichaceae*, *Ruminococcaceae*, *Lachnospiraceae*, *Streptococcaceae*, *Veillonellaceae* and *Peptostreptococcaceae*) found to correlate with disease activity in some cases
	5 HC			
Quince et al. [55] (2015)	23 CD	Stool	16S rRNA gene V4 region and shotgun metagenome NGS	Decreased diversity, decreased abundance in 34 genera (some of the most-impacted included *Bifidobacterium*, *Ruminicoccus* and *Faecalibacterium*) and increased *Lactococcus*
	21 HC			
Lewis et al. [56] (2015)	22 CD	Stool	Shotgun metagenome NGS	Decreased *Dialister*, *Dorea*, *Gordonibacter*, *Haemophilus*, *Streptococcus* and increased *Alistipes*
Schwerd et al. [54] (2016)	8 CD	Stool	16s rRNA gene, V3–V4 region NGS	Decreased abundance of phylum Bacteroidetes, including family *Bacteroidaceae*, *Porphyromonadaceae*, and *Rikenellaceae*; increased abundance of phylum Firmicutes, including *Ruminococcaceae* and *Christensenellaceae*
Guinet-Charpentier et al. [58] (2016)	4 CD	Stool	16S rRNA gene NGS	Decrease in *Escherichia-Shigella* and *Sutterella* (*Proteobacteria phylum*); increase in *Alistipes*
Dunn et al. [59] (2016)	10 CD	Stool	16s rRNA gene, V6–V8 region NGS	In patients who sustained remission (SR) after EEN, EEN reduced diversity. SR was associated with *Akkermansia muciniphila*, *Bacteroides* (incl. *B. fragilis* and *B. ovatus*), *Lachnospiraceae*, and *Ruminococcaceae* In patients who did not achieve/sustain remission (NSR), EEN increased in diversity. NSR was associated with *Bacteroides* (incl. *B. plebeius*), *Enterobacteriaceae* (incl. *Klebsiella*), and *Prevotella*
	5 HC			

CD: Crohn’s disease; DGGE: denaturing gradient gel electrophoresis; HC: healthy control; NGS: next generation sequencing; PCR: polymerase chain reaction; TGGE: temperature gradient get electrophoresis; T-RFLP: terminal restriction fragment length polymorphism.
